# Novel biomarkers distinguishing pancreatic head Cancer from distal cholangiocarcinoma based on proteomic analysis

**DOI:** 10.1186/s12885-019-5548-x

**Published:** 2019-04-05

**Authors:** Tsutomu Takenami, Shimpei Maeda, Hideaki Karasawa, Takashi Suzuki, Toru Furukawa, Takanori Morikawa, Tatsuyuki Takadate, Hiroki Hayashi, Kei Nakagawa, Fuyuhiko Motoi, Takeshi Naitoh, Michiaki Unno

**Affiliations:** 1 Department of Surgery, Tohoku University Graduate School of Medicine, 1-1 Seiryo-machi, Aoba-ku, Sendai, Miyagi 980-8574 Japan; 2 Department of Pathology and Histotechnology, Tohoku University Graduate School of Medicine, Sendai, Japan; 3 Department of Histopathology, Tohoku University Graduate School of Medicine, Sendai, Japan

**Keywords:** Pancreatic cancer, Cholangiocarcinoma, Proteomics, Biomarker, Differential diagnosis

## Abstract

**Background:**

The differentiation between pancreatic head cancer (PHC) and distal cholangiocarcinoma (DCC) can be challenging because of their anatomical and histopathological similarity. This is an important problem, because the distinction has important implications for the treatment of these malignancies. However, there are no biomarkers for the differential diagnosis of PHC and DCC. The present study aimed to identify novel diagnostic immunohistochemical biomarkers to distinguish PHC from DCC.

**Methods:**

Liquid chromatography tandem mass spectrometry (LC-MS/MS) was employed to detect candidate proteins. Ten PHC and 8 DCC specimens were analyzed by LC-MS/MS. Selected proteins were evaluated, using immunohistochemical analysis, to determine whether they would be appropriate biomarkers. Finally, we generated biomarker panels to improve diagnostic accuracy. We applied these panels to clinically difficult cases (cases in which different diagnoses were made before and after operation).

**Results:**

Consequently, 1820 proteins were detected using LC-MS/MS. Fifteen differentially expressed proteins were selected as candidates based on semi-quantitative comparison. We first performed immunohistochemical staining on samples from the small cohort group (12 PHCs and 12 DCCs) using 15 candidates. KRT17, ANXA10, TMEM109, PTMS, and ATP1B1 showed favorable performances and were tested in the next large cohort group (72 PHCs and 74 DCCs). Based on immunohistochemical analysis, KRT17 performed best for the diagnosis of PHC as a single marker; additionally, PTMS exhibited good performance for the diagnosis of DCCs. Moreover, we indicated the KRT17+/ANXA10+/PTMS- staining pattern as a biomarker panel for the correct diagnosis of PHC and KRT17−/ANXA10−/PTMS+ for the diagnosis of DCC. After immunohistochemical staining for examining samples from the clinically difficult cases, these panels showed satisfactory diagnostic performance with 85.7% (6/7) accuracy.

**Conclusions:**

We conclude that 5 proteins and 2 biomarker panels are promising for distinguishing PHC from DCC, and patients with an equivocal diagnosis would benefit from the application of these biomarkers. Confirmatory studies are needed to generalize these findings to other populations.

**Electronic supplementary material:**

The online version of this article (10.1186/s12885-019-5548-x) contains supplementary material, which is available to authorized users.

## Background

Pancreatic cancer is the fourth leading cause of cancer death in Europe and the United States with an overall 5-year survival rate of only about 7% [1–3]. Conversely, biliary tract cancer is a relatively rare disease in Western countries; however, the incidence of biliary tract cancer appears to have been increasing worldwide over the last few decades, particularly in East Asia. Biliary tract cancer is currently the sixth leading cause of death in Japan [3–5].

Oncologic therapies differ between pancreatic cancer and biliary tract cancer. Gemcitabine monotherapy had been the standard first-line treatment for patients with unresectable pancreatic cancer and the only therapeutic option for unresectable biliary tract cancer; however, the efficacy of this regimen was unsatisfactory [6]. In the last decade, the FOLFIRINOX (combination of oxaliplatin and irinotecan plus 5-fluorouracil) regimen improved overall survival and progression-free survival in patients with metastatic pancreatic cancer [7]. In addition, a combination chemotherapy consisting of nab-paclitaxel plus gemcitabine also improved prognosis [8]. On the other hand, the ABC-02 (Advanced Biliary Tract Cancer) study had established gemcitabine plus cisplatin as the standard first-line chemotherapy for patients with unresectable biliary tract cancer [9].

Biliary tract cancers are typically classified as intrahepatic cholangiocarcinoma (ICC), perihilar cholangiocarcinoma, distal cholangiocarcinoma (DCC), and gallbladder cancer [10]. In terms of pancreaticobiliary anatomy, the distal bile duct passes through the head of the pancreas. In general, the diagnosis of pancreatic head cancer (PHC) and DCC is made based on histomorphological evaluation of endoscopic biopsy before treatment [11]. The histological type of both pancreatic and biliary tract cancers is typically adenocarcinoma [12, 13]. Because of their anatomical and histopathological similarity, the distinction between PHC and DCC is sometimes difficult [14]. Patients with an equivocal diagnosis might not receive optimum chemotherapy as well as the effect expected from the treatment. Therefore, there is an urgent need to identify novel diagnostic markers for distinguishing PHC from DCC.

Mass spectrometry-based proteomics is an indispensable tool for biomarker discovery. Shotgun proteomics combines mass spectrometry (MS) and liquid chromatography (LC) to comprehensively identify proteins in complex mixtures [15]. Formalin-fixed paraffin-embedded (FFPE) tissues have routinely been archived in hospitals for a long period. We previously reported prognostic protein markers in pancreatic ductal adenocarcinoma and diagnostic protein markers in extrahepatic cholangiocarcinoma identified by proteomic approach using FFPE tissue [16–18].

Although several immunohistochemical markers have been studied to distinguish pancreatic cancer from cholangiocarcinoma, the studies primarily focused on ICC and included ICC cases in the biliary tract cancer group [19–21]. There are no immunohistochemical markers for the differential diagnosis of PHC and DCC. This study reveals novel diagnostic markers in the differentiation between PHC and DCC using shotgun proteomics of FFPE tissues.

## Methods

**Fig. 1:**
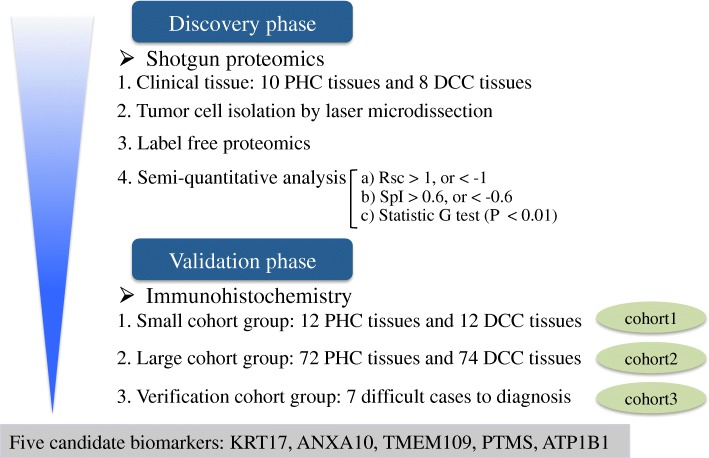
Brief workflow of this study for biomarker discovery and validation. Rsc: Protein ratio from spectral counting, SpI: Spectral index, PHC: Pancreatic head cancer, DCC: Distal cholangiocarcinoma

A brief workflow of our study is depicted in Fig. 1.

### Patient characteristics and FFPE tissue samples

We retrospectively searched patients with PHC and DCC, who underwent surgical resection between April 1998 and December 2016 in our hospital. None of the patients received radiation or neoadjuvant chemotherapy prior to surgery. ICC, perihilar cholangiocarcinoma, gallbladder cancer, and carcinoma of the ampulla of Vater were excluded. We adopted well, moderate, and poorly differentiated adenocarcinomas; rare histologic variants were not included. In total, 99 PHCs and 96 DCCs were examined. The pathological diagnosis and clinicopathological stage were determined according to the Union for International Cancer Control (UICC) 7th edition [22]. We selected 10 PHCs and 8 DCCs for proteomics. The remaining 89 PHCs and 88 DCCs samples served in the validation phase. We divided these 177 samples into three groups: small cohort group, large cohort group, and verification cohort group. The small cohort group (cohort 1) was composed of 12 PHCs and 12 DCCs for immunohistochemical screening. First, we performed immunohistochemical staining on the small cohort group and selected candidate proteins to minimize time and material consumption. The large cohort group (cohort 2), which was comprised of 72 PHCs and 74 DCCs, was used for the evaluation of diagnostic performance by immunohistochemistry. Based on immunohistochemical results of the large cohort group, we selected candidate proteins and generated biomarker panels consisting of three candidate proteins to improve diagnostic accuracy. All types of biomarker panels were evaluated in terms of sensitivity, specificity, positive likelihood ratio, and negative likelihood ratio. The remaining 5 PHC and 2 DCC cases were defined as clinically difficult cases to diagnose (cohort 3). These cases had completely opposite diagnoses between pre- and post-operation; 5 patients diagnosed as DCC preoperatively were consequently revealed as PHC by pathological findings, and vice versa. The pathologic diagnoses were carefully re-reviewed by a pathologist with an expertise in pancreaticobiliary malignancies to ensure accuracy. Finally, we applied the biomarker panels to the clinically difficult cases to verify the effectiveness of the biomarker panels.

### Laser micro-dissection and protein extraction

Target lesions were initially detected on serial sections stained with hematoxylin and eosin. For laser micro-dissection, 10-μm sections were cut onto DIRECTOR slides (Expression Pathology, Gaithersburg, MD, USA). All sections were deparaffinized three times with xylene 5 min, rehydrated with graded ethanol solutions and distilled water, stained with hematoxylin, and then air-dried. Leica LMD6000 (Leica Microsystems GmbH, Germany) was used, and about 30,000 cancer cells (8 mm^2^) were collected into the cap of a 0.2 mL polymerase chain reaction tube. Peptide extraction was performed with a Liquid Tissue MS Protein Prep kit (Expression Pathology) following manufacturer’s instructions.

### Shotgun proteomics by LC-MS/MS

All peptide-mixture samples were analyzed in LC-MS/MS using a Finnigan LXQ linear ion-trap mass spectrometer (Thermo Fisher, San Jose, CA, USA) [18, 23]. A capillary reverse phase LC-MS/MS system (ZAPLOUS System: AMR, Tokyo, Japan) composed of a Paradigm MS4 (Michrom BioResources, Auburn, CA, USA), an HTC PAL autosampler (CTC Analytics, Zwingen, Switzerland), and a Finnigan LXQ linear ion-trap mass spectrometer was equipped with an ADVANCE nanospray ionization source (Michrom BioResources).

### Data analysis and protein identification

Protein identification was performed using MASCOT software (version 2.3.02, Matrix Science, UK). The MS/MS spectral raw data were searched against *Homo sapiens* entries in the Swiss-Prot database (Release 2011.03, 20,234 entries). Peptide and fragment mass tolerants were 2.0 Da and 1.0 Da, respectively. Trypsin specificity was applied with a maximum of 2 missed cleavages. Methionine oxidation and N-formylation including formyl (K), formyl (R), and formyl (N-terminus) were considered as variable modification. A *P*-value less than 0.05 was considered significant. Reported results were obtained from triplicate LC-MS runs for each sample.

### Semi-quantitative comparison with spectral counting

The spectral counting method was utilized for comparison of protein expressions across all tissue samples. The spectral count value was determined by the number of peptide spectra with high confidence (Mascot ion score, *P* <  0.05). Fold changes in the expressed proteins on a base-2 logarithmic scale were calculated using the ratio from spectral counting (RSC) as defined by Old et al. [24]. RSC > 1 or < − 1 was consistent with fold changes > 2 or <  0.5, respectively. Spectral index (SpI) was also used to assess the differences in relative abundances of identified proteins between the 2 groups. SpI values ranged from − 1 to + 1 with values near 0 indicating similar relative peptide abundance between the 2 groups. Proteins satisfying the following conditions were candidates for the study: RSC > 1 or < − 1, SpI > 0.6 or < − 0.6, and *P* <  0.01 by the non-parametric *G*-test.

### Immunohistochemistry

FFPE samples from 177 patients were used for a validation analysis. Sections (4-μm thick) cut from the FFPE blocks were deparaffinized with xylene and rehydrated with graded alcohol followed by distilled water. For antigen retrieval, tissue slides were heated in an autoclave at 120 degrees Celsius for 5 min in a citrate acid buffer (10 mM citric acid, pH 6.0). Sections were covered with the diluted primary antibody and incubated at 4 degrees overnight. Endogenous peroxidase activity was blocked by methanol containing 0.3% hydrogen peroxidase. The labeled antigens were identified by the horseradish peroxidase EnVision System (DAKO, Glostrup, Denmark) after incubating for 60 min at room temperature. Color development was visualized with 3,3′-diaminobenzidine tetrahydrochloride (Dojindo Lab, Kumamoto, Japan). The sections were lightly counterstained with hematoxylin. Technical details for immunohistochemistry are given in Additional file 1: Table S1.

### Evaluation of immunohistochemical staining

Two authors (T. T. and T. S.) evaluated all slides of immunostained sections and scored for percentage of immunoreactive cells (labeling index). Immunoreactivity was regarded as positive if more than 10% tumor cells showed staining. Nuclear immunoreactivities for annexin A10 (ANXA10) and parathymosin (PTMS) were assessed, while cytoplasmic staining was considered positive for the other 13 proteins.

### Statistical analysis

All statistical analyses were performed using JMP software version 13.0 (SAS Institute, Cary, NC, USA). Fisher’s exact test was applied to assess the statistical significance of differences in the validation phase. To evaluate diagnostic performance, we used 2 by 2 contingency tables with binomial 95% confidence intervals.

## Results

A total of 195 cases were assessed in this study. Clinical and histopathological data for each cohort group are summarized in Table 1.

**Table 1:** Clinical and histopathological data

	MS		IHC: Small cohort group		IHC: Large cohort group		IHC: Clinically difficult cases	
	PHC	DCC	PHC	DCC	PHC	DCC	PHC	DCC
N	10	8	12	12	72	74	5	2
Sex								
Male	5	5	8	10	38	55	5	1
Female	5	3	4	2	34	19	0	1
Age								
Mean	64.5	70.1	65.4	73.4	63.1	68.6	70.2	63
Range	48–79	60–76	50–82	63–79	27–82	47–83	65–76	52–74
Histology								
Well	0	4	1	2	8	12	1	0
Moderate	10	4	9	6	61	55	4	1
Poor	0	0	1	2	3	7	0	1
UICC Stage								
I	1	4	0	2	7	24	0	0
II	8	4	9	9	52	48	5	1
III	1	0	0	0	1	0	0	0
IV	0	0	3	1	12	2	0	1

*MS* Mass spectrometry, *IHC* Immunohistochemistry, *PHC* Pancreatic head cancer, *DCC* Distal cholangiocarcinoma, *UICC* Union for international cancer control

### Protein identification by shotgun proteomics and semiquantitative comparison

In the shotgun proteomic analysis, we identified 1361 proteins in PHC and 1274 in DCC. In total, 1820 proteins were identified. The identified proteins were semi-quantitatively compared using the spectral counting method. Candidate proteins were chosen based on Rsc > 1 or < − 1, SpI > 0.6 or < − 0.6, and also statistical significance (*p* <  0.01 by nonparametric G test). We selected 18 proteins with different expression levels in the PHC and DCC groups. Shotgun proteomics revealed 5 proteins to be overexpressed in PHC as compared with in DCC, and 13 proteins to be overexpressed in DCC. Three ribosomal proteins were excluded from further analysis. Therefore, we set these 15 proteins as candidates for the validation phase (Table 2).

**Table 2:** Proteins identified in shotgun proteomics

Accession Number	Gene	Protein name	Spectral counting method				G-test	
			Spectral count		Rsc	SpI	G-score	*P*-value
			PHC	DCC				
Up-regulated in PHC								
Q9UJ72	ANXA10	Annexin A10	62	9	−2.49	0.70	39.2	< 0.01
Q04695	KRT17	Cytokeratin-17	119	20	−2.37	0.62	68.8	< 0.01
P21333	FLNA	Filamin A	124	21	−2.36	0.66	71.4	< 0.01
P06702	S100A9	S100 A9	39	6	−2.36	0.63	23.7	< 0.01
Q9BVC6	TMEM109	Transmembrane protein 109	16	2	−2.27	0.66	10.8	< 0.01
Up-regulated in DCC								
P05026	ATP1B1	Sodium/potassium-transporting ATPase subunit beta-1	0	15	3.84	−0.88	21.6	< 0.01
P21397	MAOA	Amino oxidase A	2	24	3.10	−0.70	23.7	< 0.01
P20962	PTMS	Parathymosin	0	7	2.86	−0.63	9.71	< 0.01
P02671	FGA	Fibrinogen alpha chain	2	17	2.63	−0.79	14.7	< 0.01
P55011	SLC12A2	Solute carrier family 12 member 2	0	5	2.46	−0.63	6.75	< 0.01
P04040	CAT	Catalase	2	14	2.37	−0.76	11.0	< 0.01
P11940	PABPC1	Polyadenylate-binding protein 1	1	9	2.33	−0.91	7.78	< 0.01
P31327	CPS1	Carbamoyl-phosphate synthase 1	23	104	2.27	−0.61	64.0	< 0.01
P40227	CCT6A	T-complex protein 1 subunit zeta	3	15	2.07	−0.62	9.68	< 0.01
P60842	EIF4A1	Eukaryotic initiation factor 4A-1	9	28	1.65	−0.73	12.0	< 0.01

*Rsc* Protein ratio from spectral counting, *SpI* Spectral index, *PHC* Pancreatic head cancer, *DCC* Distal cholangiocarcinoma

### Validation by immunohistochemical staining

The 15 candidates were first validated in the small cohort group including 12 PHC and 12 DCC to access cell type specific staining. Table 3 shows the positive rate for each protein in both tumor groups. Among the 15 proteins, we found the immunohistochemical results of the small cohort group were compatible with those of the shotgun proteomic analysis. We imposed the conditions to select for further evaluation; the positive rate was higher than 50% in either group and fold change between the two groups was more than 1.5-fold. ANXA10, cytokeratin-17 (KRT17), transmembrane protein 109 (TMEM109), sodium/potassium-transporting ATPase subunit beta-1 (ATP1B1), and PTMS demonstrated favorable results and were, therefore, transferred to the next validation stage using the large cohort group of 72 PHC and 74 DCC. The representative staining patterns of these 5 proteins are shown in Additional file 2: Figure S1.

**Table 3:** Immunohistochemical staining results of 15 candidate proteins

Gene	Protein name	IHC: Small cohort group			IHC: Large cohort group		
		PHC	DCC	P-value	PHC	DCC	P-value
Putatively PHC specific by MS							
ANXA10	Annexin A10	7/12 (58%)	1/12 (8.3%)	0.0272	59/72 (81.9%)	36/74 (48.7%)	0.0001
KRT17	Cytokeratin-17	10/12 (83.3%)	6/12 (50%)	0.193	55/72 (76.4%)	21/74 (28.4%)	0.0001
FLNA	Filamin A	1/12 (8.3%)	1/12 (8.3%)	1			
S100A9	S100 A9	2/12 (16.7%)	1/12 (8.3%)	1			
TMEM109	Transmembrane protein 109	9/12 (75%)	5/12 (42%)	0.214	48/72 (66.7%)	27/74 (35.4%)	0.0003
Putatively DCC specific by MS							
ATP1B1	Sodium/potassium-transporting ATPase subunit beta-1	3/12 (25%)	7/12 (58%)	0.324	30/72 (41.7%)	45/74 (60.8%)	0.0310
MAOA	Amino oxidase A	6/12 (50%)	5/12 (42%)	1			
PTMS	Parathymosin	1/12 (8.3%)	9/12 (75%)	0.0028	20/72 (27.8%)	45/74 (60.8%)	0.0001
FGA	Fibrinogen alpha chain	0/12 (0%)	2/12 (16.7%)	0.478			
SLC12A2	Solute carrier family 12 member 2	5/12 (42%)	7/12 (58%)	0.684			
CAT	Catalase	2/12 (16.7%)	4/12 (33.3%)	0.640			
PABPC1	Polyadenylate-binding protein 1	5/12 (42%)	6/12 (50%)	1			
CPS1	Carbamoyl-phosphate synthase 1	1/12 (8.3%)	4/12 (33.3%)	0.312			
CCT6A	T-complex protein 1 subunit zeta	6/12 (50%)	7/12 (58%)	1			
EIF4A1	Eukaryotic initiation factor 4A-1	4/12 (33.3%)	5/12 (42%)	1			

*MS* Mass spectrometry, *IHC* Immunohistochemistry, *PHC* Pancreatic head cancer, *DCC* Distal cholangiocarcinoma

The result of the immunohistochemical analysis in the large cohort group is described in Table 3. The diagnostic performance of candidate proteins when discriminating between PHC and DCC are listed in Table 4. All 5 proteins yielded *p* values below 0.05 in the Fisher’s exact test. KRT17 stained 76.4% of PHC and 28.4% of DCC. KRT17 provided the best diagnostic performance for the diagnosis of PHC (76.4% sensitivity, 71.6% specificity, 2.69 positive likelihood ratio, 0.33 negative likelihood ratio). Similar to KRT17, ANXA10 staining was frequently positive in PHC (81.9%) and moderately in DCC (48.7%). KRT17, ANXA10, and TMEM109 were assumed to be expressed in PHC by proteomic analysis; whereas, PTMS and ATP1B1 demonstrated an overexpression in DCC. Immunohistochemical staining of PTMS revealed more positively in DCC (60.8%) compared to PHC (27.8%). Positive staining for ATP1B1 was observed in 60.8% of DCC and 41.7% of PHC samples. PTMS exhibited better performance (60.8% sensitivity, 72.2% specificity, 2.19 positive likelihood ratio, 0.54 negative likelihood ratio) than ATP1B1.

**Table 4:** Diagnostic performances of 5 candidate proteins and biomarker panels

Target	Positive in PHC	Positive in DCC	SN	SP	LR (+) (95% CI)	LR (−)(95% CI)	P-value
Single marker for PHC diagnosis							
KRT17	55/72	21/74	76.4%	71.6%	2.69 (2.50–2.90)	0.330 (0.299–0.364)	0.0001
ANXA10	59/72	36/74	81.9%	51.3%	1.68 (1.63–1.72)	0.352 (0.303–0.408)	0.0001
TMEM109	48/72	27/74	66.7%	63.5%	1.82 (1.72–1.94)	0.525 (0.490–0.563)	0.0003
Single marker for DCC diagnosis							
PTMS	20/72	45/74	60.8%	72.2%	2.19 (2.04–2.35)	0.543 (0.520–0.566)	0.0001
ATP1B1	30/72	45/74	60.8%	58.3%	1.45 (1.39–1.54)	0.672 (0.635–0.711)	0.031
Panel for PHC diagnosis							
KRT17+/ANXA10+/PTMS-	34/72	6/74	47.2%	91.9%	5.82 (4.18–8.11)	0.574 (0.559–0.590)	0.0001
Panel for DCC diagnosis							
KRT17−/ANXA10−/PTMS+	2/72	17/74	23.0%	97.2%	8.27 (3.46–19.7)	0.792 (0.786–0.798)	0.0003

*PHC* Pancreatic head cancer, *DCC* Distal cholangiocarcinoma, *SN* Sensitivity, *SP* Specificity, *LR* (+) Positive likelihood ratio, *LR* (−) Negative likelihood ratio, *CI* Confidence interval

### Combination of candidate proteins for biomarker panels

In addition to single diagnostic markers, we generated biomarker panels consisting of our candidate proteins to improve diagnostic accuracy. As the result of the staining patterns in the large cohort group, PHC exhibited a KRT17+/ANXA10+/PTMS- staining pattern, which was seen in 34 cases (47.2%). Six of 74 DCC cases (8.1%) showed this combinatorial pattern. When used for the diagnosis of PHC, the sensitivity, specificity, and positive/negative likelihood ratio were 47.2, 91.9% and 5.82/0.574, respectively (Table 4). In contrast, DCC had a tendency to show a KRT17−/ANXA10−/PTMS+ staining profile in 17 cases (23.0%). Of 72 PHC cases, this immunoreactive pattern was demonstrated in only 2 cases (2.8%). The sensitivity, specificity, and positive/negative likelihood ratio of the KRT17−/ANXA10−/PTMS+ immunophenotype were 23.0, 97.2%, and 8.27/0.792, respectively, for the diagnosis of DCC (Table 4). These panels yielded low sensitivity, but high specificity compared with single diagnostic markers.

### Immunohistochemical verification for biomarker panels in clinically difficult cases

**Fig. 2:**
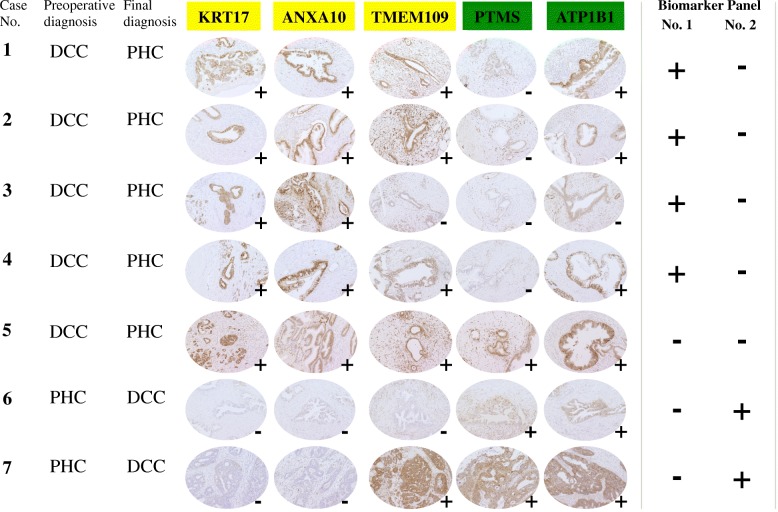
Immunohistochemical verification for 2 biomarker panels in cohort 3. PHC: Pancreatic head cancer, DCC: Distal cholangiocarcinoma, Original magnification: × 200. Panel No.1: KRT17+/ANXA10+/PTMS-, Panel No.2: KRT17−/ANXA10−/PTMS+

We selected 5 PHC and 2 DCC clinically difficult cases, which had opposite diagnoses between preoperative investigations and pathological findings. We applied 5 candidate proteins to these cases to achieve correct diagnoses (Fig. 2). KRT17+/ANXA10+/PTMS- panel diagnosed correctly 4 of 5 PHC cases, which were considered as DCC in preoperative imaging; whereas, KRT17−/ANXA10−/PTMS+ panel diagnosed 2 of 2 DCC cases correctly.

## Discussion

Pancreatic and biliary tract cancers are aggressive malignancies with poor prognoses and low survival rates [1, 3, 4]. The distal bile duct is located within the head of the pancreas. On account of their close anatomical proximity and histopathological similarity, it is sometimes difficult to distinguish PHC from DCC [14]. The distinction between PHC and DCC has important implications for patient management, especially in chemotherapy [7–9]. Thus, it is essential to find new diagnostic markers for the distinction between PHC and DCC.

Several studies have identified protein markers to discriminate pancreatic adenocarcinoma from cholangiocarcinoma. Ney et al. suggested podocalyxin-like protein 1 (PODXL-1) as a useful biomarker to differentiate pancreatic adenocarcinoma from biliary adenocarcinoma [21]. Their immunohistochemical analysis demonstrated the expression of PODXL-1 in 44% (71/160) of pancreatic adenocarcinomas, whereas none (0/18) of the intrahepatic and one (1/13) of the extrahepatic were stained. Hooper et al. combined two proteins, human pancreatic cancer fusion 2 (HPC2) and N-cadherin, for an immunohistochemical differential biomarker panel [19]. HPC2 staining was observed in 80% (48/60) of pancreatic cancers and 32% (10/31) of cholangiocarcinomas; whereas, N-cadherin stained 27% (16/60) of the pancreatic cancers and 58% (18/31) of cholangiocarcinomas. They developed a biomarker panel composed of both proteins, and improved the specificity and positive likelihood ratios. A biomarker panel composed of four proteins (S100P, pVHL, MUC5AC and KRT17) was reported to be helpful in discriminating between primary ICC and metastatic PDAC by Lok et al. [20]. However, their studies adopted intrahepatic cases, for the most part, and set them into the cholangiocarcinoma group.

In the current study, we employed a shotgun MS approach, which has advantages in the detection of low abundance proteins with broad proteome coverage to effectively detect biomarker candidates for the discovery phase [25]. In clinical practice, pathological and immunohistochemical studies are essential for the final diagnosis in detecting pancreaticobiliary malignancy. The majority of pancreatic and biliary tract neoplasms is histologically classified into tubular adenocarcinoma, which shows similar morphological features regardless of the origin [12, 13]. Therefore, immunohistochemical analysis plays a key role in the final diagnosis, and, so, we adopted immunohistochemical staining for the validation phase in this study. Through this framework, we identified 5 proteins as biomarkers in distinguishing PHC from DCC and strongly suggest that KRT17, ANXA10, TMEM109, PTMS, and ATP1B1 are promising biomarkers.

Among these proteins, KRT17, ANXA10, and TMEM109 were supposed to be good markers for the diagnosis of PHC. Unfortunately, so far, there are no reports on the function of human TMEM109. However, in several studies, it has been suggested that KRT17 and ANXA10 are related to pancreatic cancer. KRT17 is a basal/myoepithelial cell keratin and is generally expressed in normal human epithelia, such as salivary glands, prostate, and breast [26, 27]. Studies have demonstrated that the expression of KRT17 might be a useful marker in differentiating pancreatobiliary adenocarcinoma from extra-pancreatobiliary adenocarcinoma [28–30]. Chu et al. reported 38 of 46 pancreatic cancer cases (83%) were positive for KRT17 and 17 of 24 (71%) ICC [28]. The immunohistochemical positive rate in pancreatic cancer is in line with our study (76%), but the positive rate in DCC slightly differs from our study (28%), partly because of genomic differences in DCC and ICC [31, 32]. KRT17 was able to discriminate between two groups with a sensitivity of 76.4% and specificity of 71.6%, and provided the best performance for the diagnosis of PHC among candidate proteins.

The annexin family is a calcium and phospholipid binding protein, which plays important roles in multiple physiological processes, such as differentiation and proliferation [33, 34]. Several annexins have been reported to associate with tumorigenesis [35]. Previous studies showed annexin A10 expression in the normal gastric mucosa and gastric adenocarcinoma [36, 37]. Lu et al. evaluated the expression in both noncancerous and primary carcinomas of major organs [38]. The authors demonstrated that ANXA10 was expressed in 78% pancreatic adenocarcinoma and 51% extrahepatic cholangiocarcinoma. Both immunohistochemical results were almost in line with our analysis. Moreover, they reported that no expression of ANXA10 was observed in the normal bile duct and pancreatic duct, and indicated that the expression of ANXA10 is an early event in the development of pancreatic and biliary adenocarcinoma. By using the MS-based proteomic analysis, Padden et al. reported that ANXA1 and ANXA10 are promising biomarkers distinguishing ICC from pancreatic cancer by immunohistochemistry [39]. In the latest paper by the same authors, they demonstrated the diagnostic performance of 14 immunohistochemical markers, including ANXA10 and KRT17, to distinguish ICC from hepatic metastases of pancreatic ductal adenocarcinoma and concluded ANXA10 had the highest diagnostic potential of all single markers [40].

On the other hand, PTMS and ATP1B1 were expected to be profitable markers for the diagnosis of DCC. PTMS is a small nuclear acidic protein that works as a co-activator of the glucocorticoid receptor [41, 42]. In addition, it also affects the transcriptional activity of NF-Kappa B, which plays important roles in the inflammatory pathway; however, little is known about PTMS functions in cancer [42]. Xin-Zhang et al. reported that PTMS was differentially expressed in nasopharyngeal carcinoma versus the adjacent non-tumor tissue using isobaric tags for the relative and absolute quantification (iTRAQ) method [43]. In the current study, PTMS provided good diagnostic performance for the diagnosis of DCC with a sensitivity of 60.8% and specificity of 72.2%.

Na+/K + -ATPase is an integral membrane protein essential for cellular osmotic regulation and maintenance of the electrochemical gradients [44, 45]. ATP1B1 encodes the beta 1 subunit of Na+/K + -ATPase. In a recent study, an overexpression of ATP1B1 was shown to predict a poor prognosis in cytogenetically normal acute myeloid leukemia and could be an unfavorable prognostic biomarker [46].

To enhance the diagnostic efficiency, we generated optimal immunohistochemical panels composed of KRT17, ANXA10, and PTMS. The KRT17+/ANXA10+/PTMS- immunophenotype demonstrated better performance for the diagnosis of PHC than any other biomarker panels and achieved a sensitivity of 47.2% and specificity of 91.9%. On the other hand, the KRT17−/ANXA10−/PTMS+ staining pattern had good performance for the diagnosis of DCC. This panel improved the specificity to 97.2% for the DCC diagnosis, although it showed a sensitivity of 23.0%. Specificity is more important than sensitivity for differential diagnoses, and it is extremely difficult to distinguish PHC from DCC in certain patients for which these panels will be of clinical value given the high specificity.

Finally, we performed immunohistochemical verification for these biomarker panels using clinically difficult cases, which had opposite diagnoses between pre- and post-operation. The KRT17+/ANXA10+/PTMS- panel was detected in 4 of 5 PHC; whereas, the KRT17−/ANXA10−/PTMS+ panel was detected in 2 DCC. The panels showed satisfactory diagnostic performance with 85.7% accuracy.

When evaluated individually, patient number 1 was a case with biliary stricture and no obvious tumor in the head of the pancreas. The patient presented with painless jaundice. Contrast-enhanced computed tomography (CT) showed intrahepatic and extrahepatic bile duct dilatation down to the superior border of the pancreas, but no obvious pancreatic mass was seen. Endoscopic retrograde cholangiopancreatography confirmed an obstruction in the distal bile duct with biliary brush cytology positive for malignancy. Therefore, the patient was diagnosed with DCC preoperatively and underwent resection. However, pathological findings of the specimen revealed pancreatic ductal adenocarcinoma that involved the distal bile duct. In general, typical imaging of pancreatic ductal adenocarcinoma demonstrates a low-density hypovascular mass on a CT scan obtained in the appropriate phase [47]. In some cases, however, CT scan shows an ambiguous iso-density lesion which is insufficient to diagnose. The application of our panel might be more appropriate in patients with atypical imaging or in cases of contrast allergy.

This study has several limitations. First, this study was performed in a Japanese-only patient cohort. Further studies in different ethnic groups and geographical locations are needed to generalize our findings. Second, the sensitivities and specificities of the biomarker panels are based on tumors with previously known histologic diagnoses. A prospective study applying these algorithms to neoplasms of unknown origin is needed to validate the diagnostic performance of our candidate proteins. Third, this study utilized solely surgically resected tumor specimens. In clinical practice, the definitive diagnosis of pancreaticobiliary malignancies rely primarily on samples obtained by endoscopic ultrasound-guided fine needle aspiration biopsy or transpapillary forceps biopsy [11, 48]. Confirming the utility of these proteins in biopsy samples will allow our biomarker panel to become useful and valuable for patients with pancreaticobiliary malignancy in the clinical setting.

## Conclusions

We performed a proteomic analysis with archival FFPE to discover novel biomarkers, which can discriminate PHC from DCC. KRT17, ANXA10, TMEM109, PTMS, and ATP1B1 were identified as candidate proteins for diagnostic biomarkers. With respect to single biomarkers, KRT17 provided the best performance for the diagnosis of PHC, whereas PTMS showed good performance for the diagnosis of DCC. Furthermore, we demonstrated the KRT17+/ANXA10+/PTMS- and KRT17−/ANXA10−/PTMS+ immunophenotypes can be promising biomarker panels for the diagnosis of PHC and DCC, respectively.

## Additional files

Additional file 1: **Table S1.** Antibodies used for current study. (DOC 45 kb)

Additional file 2: **Figure S1.** Representative immunostaining pattern of 5 candidate proteins. Original magnification: × 200, Scale bars represent 100 μm. (PDF 212 kb)

## Abbreviations

ANXA10: annexin A10
ATP1B1: sodium/potassium-transporting ATPase subunit beta-1
CT: computed tomography
DCC: distal cholangiocarcinoma
FFPE: formalin-fixed paraffin-embedded
ICC: intrahepatic cholangiocarcinoma
KRT17: cytokeratin-17
LC: liquid chromatography
MS: mass spectrometry
MS/MS: tandem mass spectrometry
PHC: pancreatic head cancer
PTMS: parathymosin
TMEM109: transmembrane protein 109
