# Supplementary material for: Novel biomarkers distinguishing pancreatic head Cancer from distal cholangiocarcinoma based on proteomic analysis
Source: BMC Cancer. 2019 Apr 5;19:318. doi: 10.1186/s12885-019-5548-x (PMC6451218; doi:10.1186/s12885-019-5548-x)
Supplement: Supplementary file 1 — Table S1. Antibodies used for current study. (DOC 45 kb) [file 12885_2019_5548_MOESM1_ESM.doc]

Supplemental Table. 1 Antibodies used for current study

| **Antibody** | **Protein name** | **Manufacture** | **Clone** | **Dilution** | **DAB** | **Positive control** | **HIER** | **Location of evaluation** |
| --- | --- | --- | --- | --- | --- | --- | --- | --- |
| ANXA10 | Annexin A10 | Nobus Biologicals, NBP1-90156 | Polyclonal | 1:500 | 12min | Stomach | pH6.0, Autoclave | Nucleus |
| FLNA | Filamin A | Sigma, HPA002925 | Polyclonal | 1:200 | 10min | Esophagus | pH6.0, Autoclave | Cytoplasm |
| S100A9 | S100 A9 | Abcam, ab92507 | Monoclonal | 1:500 | 7min | Spleen | pH6.0, Autoclave | Cytoplasm |
| TMEM109 | Transmembrane protein 109 | Abcam, ab121349 | Polyclonal | 1:500 | 15min | Spleen | pH6.0, Autoclave | Cytoplasm |
| KRT17 | Cytokeratin-17 | Sigma, HPA000452 | Polyclonal | 1:200 | 15min | Prostate | pH6.0, Autoclave | Cytoplasm |
| EIF4A1 | Eukaryotic initiation factor 4A-I | Abcam, ab31217 | Polyclonal | 1:200 | 10min | Colon | pH6.0, Autoclave | Cytoplasm |
| PABPC1 | Polyadenylate-binding protein 1 | Thermo Fisher, PA5-29883 | Polyclonal | 1:100 | 10min | Esophagus | pH6.0, Autoclave | Cytoplasm |
| CCT6A | T-complex protein 1 subunit zeta | Thermo Fisher, PA5-29883 | Polyclonal | 1:200 | 15min | Colon | pH6.0, Autoclave | Cytoplasm |
| CPS1 | Carbamoyl-phosphate synthase 1 | Sigma, HPA021400 | Polyclonal | 1:200 | 15min | Liver | pH6.0, Autoclave | Cytoplasm |
| SLC12A2 | Solute carrier family 12 member 2 | Sigma, HPA020130 | Polyclonal | 1:500 | 10min | Stomach | pH6.0, Autoclave | Cytoplasm |
| CAT | Catalase | Nobus Biologicals, NBP2-24916 | Polyclonal | 1:500 | 10min | Liver | pH6.0, Autoclave | Cytoplasm |
| FGA | Fibrinogen alpha chain | Sigma, HPA064755 | Polyclonal | 1:500 | 15min | Kidney | pH6.0, Autoclave | Cytoplasm |
| PTMS | Parathymosin | Sigma, HPA038186 | Polyclonal | 1:250 | 15min | Colon | pH6.0, Autoclave | Nucleus |
| MAOA | Amino oxidase A | Nobus Biologicals, NBP1-19796 | Polyclonal | 1:300 | 15min | Colon | pH6.0, Autoclave | Cytoplasm |
| ATP1B1 | Sodium/potassium-transporting ATPase subunit beta-1 | Sigma, HPA012911 | Polyclonal | 1:200 | 15min | Colon | pH6.0, Autoclave | Cytoplasm |

HIER: heat induced epitope retrieval
