# Supplementary figures and images for: Novel biomarkers distinguishing pancreatic head Cancer from distal cholangiocarcinoma based on proteomic analysis
Source: BMC Cancer. 2019 Apr 5;19:318. doi: 10.1186/s12885-019-5548-x (PMC6451218; doi:10.1186/s12885-019-5548-x)

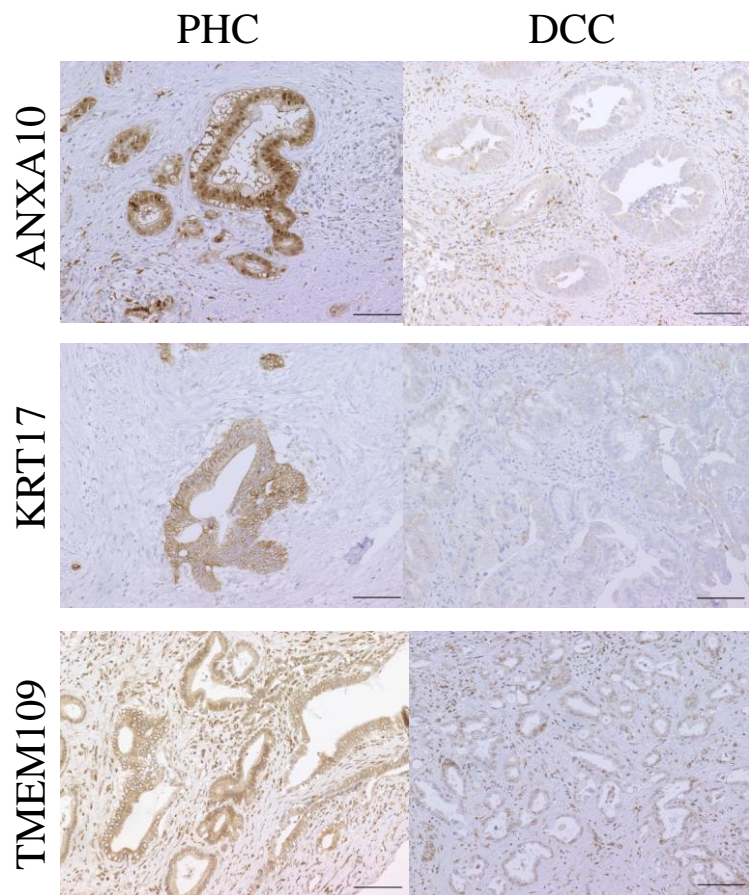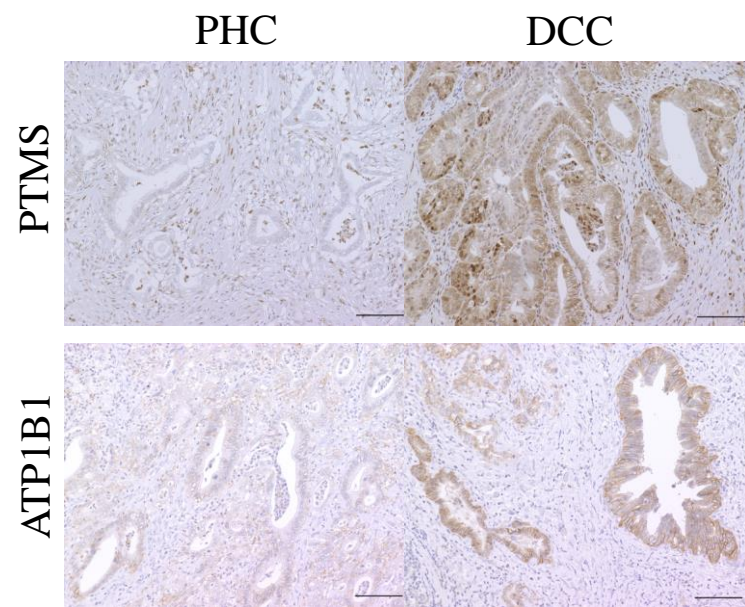

Supplement: Supplementary file 2 — Figure S1. Representative immunostaining pattern of 5 candidate proteins. Original magnification: × 200, Scale bars represent 100 μm. (PDF 212 kb) [file 12885_2019_5548_MOESM2_ESM.pdf]
